# Supplementary figures and images for: Cisnormativity as a structural barrier to STI testing for trans masculine, two-spirit, and non-binary people who are gay, bisexual, or have sex with men
Source: PLoS One. 2022 Nov 28;17(11):e0277315. doi: 10.1371/journal.pone.0277315 (PMC9704602; doi:10.1371/journal.pone.0277315)

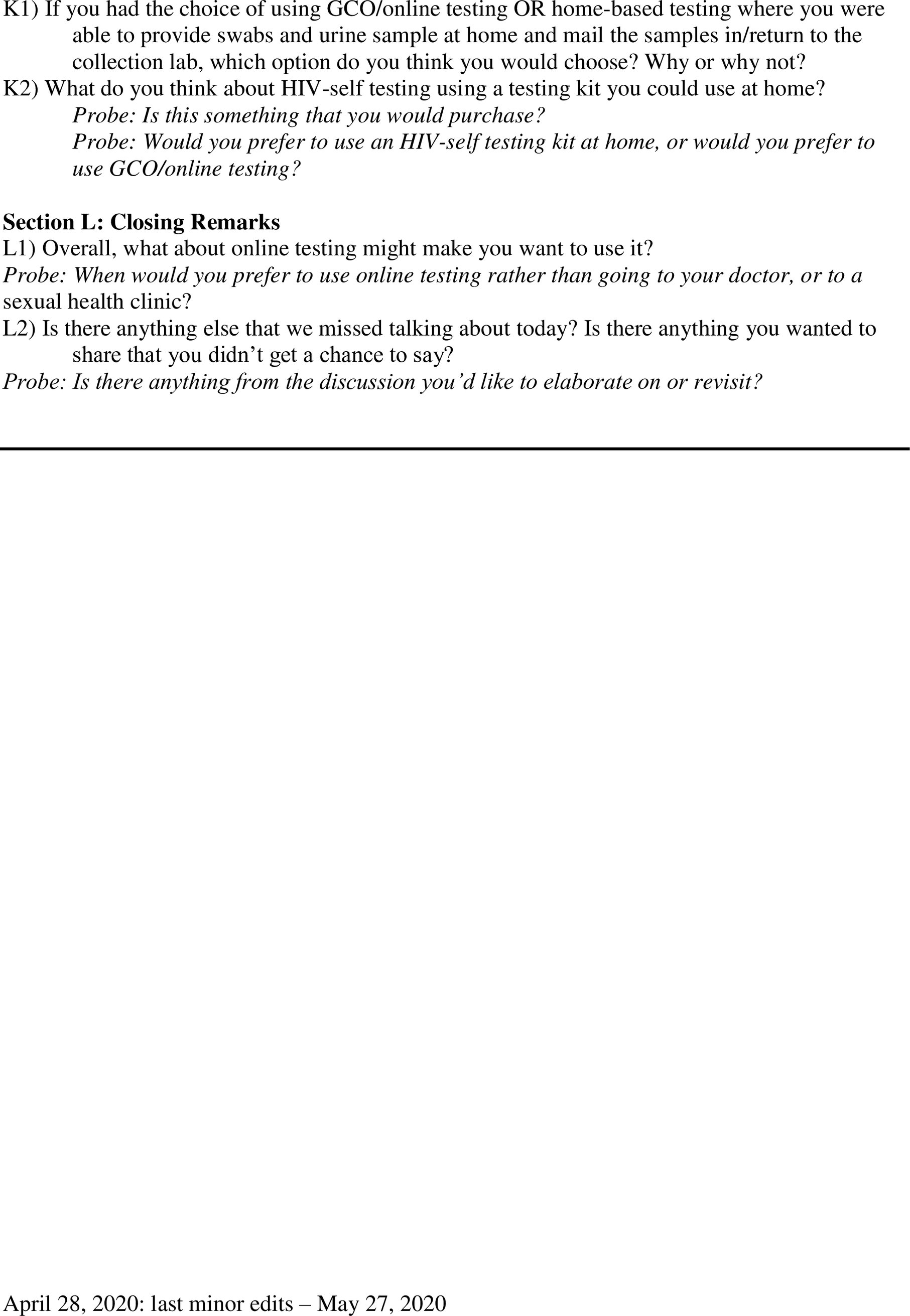

Supplement: S1 Fig — (TIF) [file pone.0277315.s001.tif]
